# Hybrid Sol-Gel Coatings Doped with Non-Toxic Corrosion Inhibitors for Corrosion Protection on AZ61 Magnesium Alloy

**DOI:** 10.3390/gels8010034

**Published:** 2022-01-05

**Authors:** Luis Rodríguez-Alonso, Jesús López-Sánchez, Aida Serrano, Oscar Rodríguez de la Fuente, Juan Carlos Galván, Noemí Carmona

**Affiliations:** 1 Departamento de Física de Materiales, Facultad de Ciencias Físicas, Universidad Complutense de Madrid, Plaza Ciencias sn, 28040 Madrid, Spain; luisro03@ucm.es (L.R.-A.); osrodrig@ucm.es (O.R.d.l.F.); 2 SpLine, Spanish CRG BM 25 Beamline, ESRF—The European Synchrotron, 71 Av. Des Martys, 38000 Grenoble, France; jesus.lopez@ucm.es; 3 Instituto de Ciencia de Materiales de Madrid (ICMM-CSIC), C/Sor Juana Inés de la Cruz, 3, 28049 Madrid, Spain; 4 Instituto de Cerámica y Vidrio (ICV-CSIC), C/Kelsen 5, Campus de Cantoblanco, 28049 Madrid, Spain; aida.serrano@icv.csic.es; 5 Centro Nacional de Investigaciones Metalúrgicas (CENIM-CSIC), Avda. Gregorio del Amo 8, 28040 Madrid, Spain; jcgalvan@cenim.csic.es

**Keywords:** sol-gel, corrosion inhibitors, L-cysteine, Hanks’ solution, AZ61 magnesium alloy

## Abstract

Physiological human fluid is a natural corrosive environment and can lead to serious corrosion and mechanical damages to light Mg–Al alloys used in prosthetics for biomedical applications. In this work, organic–inorganic hybrid coatings doped with various environmentally friendly and non-toxic corrosion inhibitors have been prepared by the sol-gel process for the corrosion protection of AZ61 magnesium alloys. Effectiveness has been evaluated by pH measurements, optical microscopy, and SEM during a standard corrosion test in a Hanks’ Balanced Salt Solution. The results showed that the addition of an inhibitor to the sol-gel coating can improve significantly the corrosion performance, being an excellent barrier for the L-cysteine-doped hybrid sol-gel films. The incorporation of TiO_2_ nanoparticles, 2-Aminopyridine and quinine organic molecules slowed down the corrosion rate of the Mg–Al alloy. Graphene oxide seemed to have the same response to corrosion as the hybrid sol-gel coating without inhibitors.

## 1. Introduction

The search for new metal alloys to be used in biomedicine has developed greatly in recent years. Among the properties, these new materials must stand out, i.e., good mechanical and elastic resistance, or at least similar to those of materials to be replaced (e.g., bone), good resistance to corrosion, and sufficiently high biocompatibility to avoid rejection [1,2].

The most frequently used alloys today are Ti-based alloys [3,4] The widespread use of titanium and its alloys as a biomaterial in the manufacture of implants is due to the fact that they meet the requirements of biocompatibility, osseointegration, mechanical properties, corrosion resistance, processability, and availability. The high level of osseointegration is because when they are implanted in hard tissue, the bone is able to grow in direct contact with the implant, without an appreciable soft tissue capsule around it. Studies have also been carried out on new alloys, such as Mg–Al alloy. This material is abundant in the Earth’s crust, and the raw material has a reasonable cost. However, its processing requires certain precautions that make the manufacture of the alloy more expensive, so that the final cost is halfway between those of stainless steel and titanium alloys [5].

The use of magnesium in medical applications has been envisioned since the middle of the last century. However, its application has recently undergone an enormous increase, mainly due to its unique properties, which, together with its low weight, make it a suitable material for use in the field of bone implants [6]. One of the characteristics of this material is that its mechanical properties are similar to those of the bone, which is not the case with other materials used in orthopedic applications, such as titanium and stainless steel. This advantage allows better natural bone recovery processes. Thus, magnesium is a biocompatible and non-toxic material, allowing the development of bio-absorbable implants, which can be regulated in their time of permanence in the body to fulfill a certain function of recovery of a tissue and later degrade naturally [7,8]. The disadvantage of magnesium-containing alloys is their high reactivity. Therefore, it is necessary to apply some type of treatment or coating to improve their corrosion resistance [9].

Several techniques have been employed to reduce the corrosion rate of reactive alloys such as Mg, for example laser shock processing [10], layer-by-layer deposition [11], electrospinning [12], or the sol-gel method. Specifically, among the possible coatings that can be used to protect Mg-based alloys such as Mg–Al alloys from degradation, the sol-gel process stands out as a very versatile, environmentally friendly and economical method of synthesis that has undergone significant development in recent years. Additionally, by this procedure, it is possible to synthesize bioactive materials only by adding functional groups such as Si–OH, Ti–OH, and Zr–OH as precursors [13]. The sol-gel process is a very well-known synthetic route consisting in three fundamental stages, i.e., hydrolysis, polycondensation, and thermal densification. Starting from a colloidal suspension of very small particles in a liquid, it develops to a gel with a two-phase solid–liquid structure. The precursors used in the sol-gel process can be organic or inorganic. Among the numerous advantages of this technique, the following stand out: the high purity of the products is obtained due to the high control in the composition of the precursor materials; there is no need of a pretreatment to cover the metal surface; the synthesis can be carried out at room temperature and at atmospheric pressure; the process has low toxicity and is economical; coatings present good chemical resistance, constituting a powerful inhibitor of the corrosion in metals and alloys. Moreover, the nanoporosity of sol-gel coatings allows them to host other molecules that can preserve their properties and thus develop new potential applications, for example, as active corrosion protection methods [14,15]. 

Several corrosion inhibitors have been studied to improve the corrosion resistance of Mg–Al alloys. The first ones studied are insoluble salts (e.g., Ce^3+^ [16], La^3+^ [17], or Zr^3+^ ions [18]), then organic molecules and their derivatives that are less toxic and have better biodegradability. Most of the first organic inhibitors are weak acids, and some of the ones used lately are molecules containing various heteroatoms, such as aminoacids [19,20]. The use of polymers such as polyaniline or polypyrrole as conducting coatings has been also tested. Here, the mechanism for protection is not due to a barrier effect, but instead, it is believed that a passive oxide film is formed on the metal surface through an anodization process [21], giving the resulting metal/coating system self-healing properties and active corrosion protection [22,23,24]. Several types of nanoparticles (NPs) have been also tested [25,26]. TiO_2_ NPs have shown to improve anticorrosion and antibacterial properties [27]. Graphene and its oxide nanosheets have been also used in sol-gel coatings due to their specific surface areas and impermeability for ions, water, and oxygen. However, they do not provide an active protection to the substrate [28,29]. 

The aim of this work is to evaluate the corrosion protection of several hybrid sol-gel coatings doped with various environmentally friendly and non-toxic corrosion inhibitors deposited on an AZ61 Mg–Al alloy, in order to improve its low resistance to corrosion. Corrosion inhibitors such as L-cystine, dimethyl glyoxine, quinine, 2-Aminopyridine, graphene sheets, and TiO_2_ NPs have been tested. The in vitro biodegradation and corrosion protection behaviors of the resulting hybrid sol-gel coatings have been evaluated by an immersion test in a Hanks’ Balanced Salt Solution (HBSS) simulating the body fluid.

## 2. Experimental

### 2.1. Synthesis of the Coatings by the Sol-Gel Method

The magnesium–aluminium (Mg–Al) alloy, commercially known as AZ61, was used as the substrate for the sol-gel coatings. It was composed of 6% by mass Al, 0.7% by mass Zn, 0.3% by mass Mn, and the rest up to 100% by mass Mg. 

Tetraethyl orthosilicate (TEOS) and 3-(Trimethoxysilyl)propyl methacrylate (MEMO) were used as the precursors of sol-gel coatings (molar ratio, 1:1). Absolute ethanol (CH_3_CH_2_OH; Sigma-Aldrich) was employed as a solvent. Distilled water was added to promote the hydrolysis, and nitric acid was added as a catalyst (HNO_3_; 70%; Sigma-Aldrich,). The molar concentrations of alkoxides, ethanol, and HNO_3_ were 1:4:0.05, respectively. 

**Figure 1:**
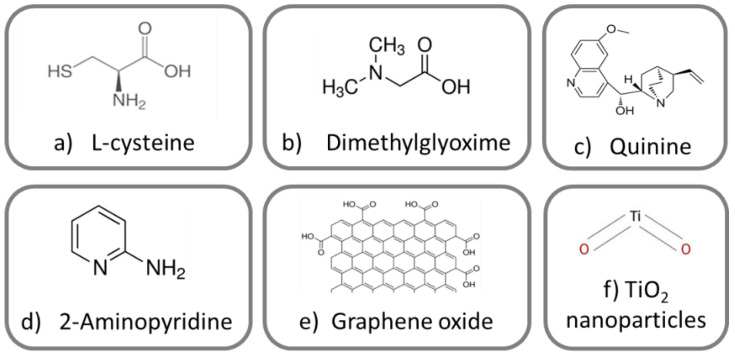
Corrosion inhibitors added into the sol-gel coatings: (**a**) L-cysteine; (**b**) dimetyl glyoxime; (**c**) quinine; (**d**) 2-Aminopyridine; (**e**) graphene oxide sheet; and (**f**) TiO_2_ nanoparticles.

Prior to the hydrolysis of the sol, six different environmentally friendly substances were used as corrosion inhibitors, i.e., L-cysteine, dimethyl glyoxime, quinine, 2-Aminopyridine, graphene sheets [30], and TiO_2_ NPs, at a 0.02% weight/volume ratio (see Figure 1). 

They were chosen due to their properties as biocompatible molecules, complexing agents, or environmentally friendly NPs. Specifically, molecules with functional groups of the type amine, carbonyl, alcoholic groups, or conjugated bonds were selected, because they are more effective corrosion inhibitors [31].

Once the sol-gel coatings were prepared with the inhibitors, they were applied to AZ61 sheets with a 30 mm × 30 mm × 3 mm size by dip-coating. The withdrawal speed was 5 mm·s^−1^. A final thermal treatment was performed at 60 °C for 3 days to complete the densification of the resulting coatings.

To evaluate the effectiveness of the coatings doped with several molecules after their application to the Mg alloy, a corrosion test with an HBSS was used. This is an aqueous solution widely used in in vivo tests that simulates physiological saline media. The samples were immersed for 14 days into the solution according to Standard ASTM G31 [32,33], and the pH was monitored every 24 h. A Crison pH25+ digital laboratory portable pH-meter was used for the data recording. After the 14-day test, the samples were removed from the solution and washed with distilled water 3 times. Then, they were left to be dried in the air and stored in a desiccator to prevent the corrosion of the surfaces.

### 2.2. Samples Characterization Techniques 

The macroscopic information of the coatings surfaces on the Mg–Al alloy before and after the degradation test was obtained by optical microscopy in the reflection mode with an Olympus BX60M optical microscope.

The surface characterization and semi-quantitative chemical composition analyses of the corroded surfaces and small deposits that remained adhered to the surface of the samples were carried out by SEM with an electronic microscope model JEOL JSM 6335F operating at an acceleration voltage of 20 kV. An energy-dispersive X-ray detector was employed for the elemental analysis of the surfaces. All samples were previously coated with 5 nm of Au to avoid charging effects. 

## 3. Results and Discussion

### 3.1. Samples Preparation

Sol-gel coatings were prepared by mixing TEOS and MEMO at a molar ratio of 1:1. Absolute ethanol was added as a solvent in a solution with the selected corrosion inhibitor, and nitric acid was added as a catalyst. Stoichiometric quantities of water were added to perform the complete hydrolysis and polycondensation. The obtained sol was magnetically stirred for 24 h, and the final pH was measured. All sols had an acid pH around 1–3, except the ones with quinine and 2-aminopyridine at pH values around 8–9.

The obtained sols were finally applied by dip-coating as explained in the experimental section, and a densification treatment was performed before the characterization of the samples. The coating thickness was obtained from the interference of transmittance spectra in the ultraviolet-visible and near-infrared regions according to [34]. All coatings resulted in thickness values below 100 ± 30 nm, except for the sample A-SG-DMG with a thickness value of 592 ± 99 nm. The descriptions of all the prepared samples are presented in Table 1.

### 3.2. Weathering Test

**Figure 2:**
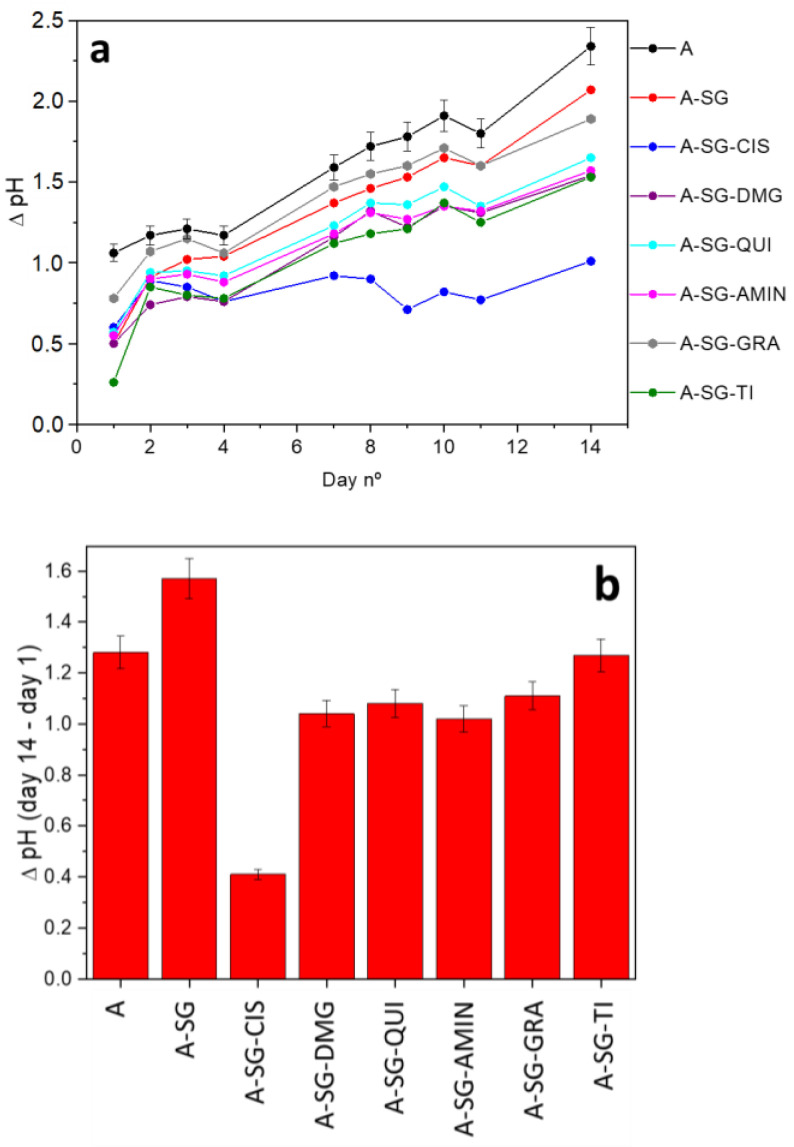
(**a**) Increase in pH of the solution versus the day of the test for samples A, A-SG, A-SG-GRA, A-SG-QUI, A-SG-AMIN, A-SG-DMG, A-SG-TI, and A-SG-CIS; (**b**) pH gradient measured for the 14 days of test in a Hanks’ Balanced Salt Solution (HBSS).

In order to explore the corrosion behavior of the coated surfaces, the samples were placed in closed containers containing approximately 150 mL of a commercial HBSS at room temperature for 14 days, as explained in the experimental section. The pH of each solution was measured every day. The pH of the Hanks’ solution itself (pH = 7.23) was measured at the beginning of each day’s measurement as a reference. The results of the variation of these pH measurements for all samples are shown in Figure 2.

For all cases, the increases in pH for all solutions during the 14 days of test were measured, reaching the maximum value at day 14. The dynamic process of the corrosion depended on the inhibitor incorporated into the coating identifying the largest pH difference for the sample A-SG in the 14 days, while the lowest gradient was observed for sample A-SG-CIS. The rest of samples showed pH gradients in the 14 days very similar between each other and within the error of the measurement.

The increases in the pH of the samples were due to the fact that the Hanks’ solution acted as a biological fluid, which caused corrosion over the time. The corrosion mechanism of Mg-based alloys is well-known, and the following reactions take place in the presence of water:

Anodic reaction: Mg_(s)_ → Mg^2+^_(ac)_ + 2e^−,^ (1)

Cathodic reaction: 2H_2_O + 2e^−^ → H_2(g)_ + 2 OH^−^_(ac)_. (2)

The obtained OH^–^ ions were responsible of the pH increase of the Hanks’ solution during the 14 days of test. In the first week, the pH values of all solutions increased considerably, and it was in the second week where three different behaviors were observed that the coatings were divided in three different groups:

(i) The solutions of the uncoated alloy (sample A), the sample coated with the hybrid sol-gel without corrosion inhibitors (sample A-SG), and the sol-gel coating with graphene oxide as an inhibitor (sample A-SG-GRA) showed increasing pH values throughout the test for 14 days. Thus, it indicated that both the uncoated alloy and the alloy coated with the blank sol-gel deteriorated throughout the test in the same way. This behavior is particularly interesting, because it indicated that a hybrid sol-gel coating without inhibitors was not effective as a protective layer against corrosion.

The increment of pH for the graphene oxide-doped sol-gel coating indicated that the barrier effect of the graphene sheets did not act enough, perhaps because the concentration of the inhibitor added was not the optimal one, and other concentrations should be researched. 

(ii) Samples A-SG-QUI, A-SG-AMIN, A-SG-DMG, and A-SG-TI stabilize pH values after two days of the test, and then, the increment of pH increased very slowly. This is probably due to the fact that the coating was very thin and nanoporous, so it did not exert a barrier effect from the beginning but allowed some corrosion of the alloy that acted as a substrate until a point was reached, where the coating inhibitors acted and protected against corrosion. This happened from the 7th day until the end of the test.

(iii) Finally, the solution containing sample A-SG-CIS showed an increase in pH during the first two days, and from the third day onwards, its pH value stabilized or decreased progressively, which was more similar to the behavior of a self-repairing coating. The increase of pH for this sample was less than 0.75 pH after the 14 days test.

Therefore, the corrosion behavior depended significantly on the inhibitor incorporated in the coating, and the effect was clearly reflected on the coating surfaces.

**Figure 3:**
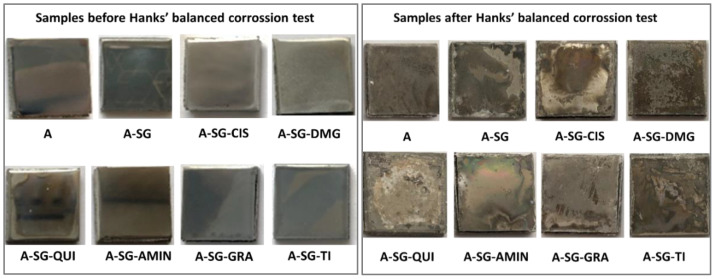
Representative macroscopic surfaces of the Mg–Al alloys substrates before and after immersion in an HBSS for 14 days for samples A, A-SG, A-SG-CIS, A-SG-DMG, A-SG-QUI, A-SG-AMIN, A-SG-GRA, and A-SG-TI. All square pieces had a size of 3 mm × 3 cm.

Figure 3 shows the photographs of the dried sample surfaces after the HBSS corrosion test. It can be seen how the corrosion affected the surface layer of the coating macroscopically and, in some cases, the deterioration reached the inner part of the alloy.

### 3.3. Optical Microscopy Characterization

**Figure 4:**
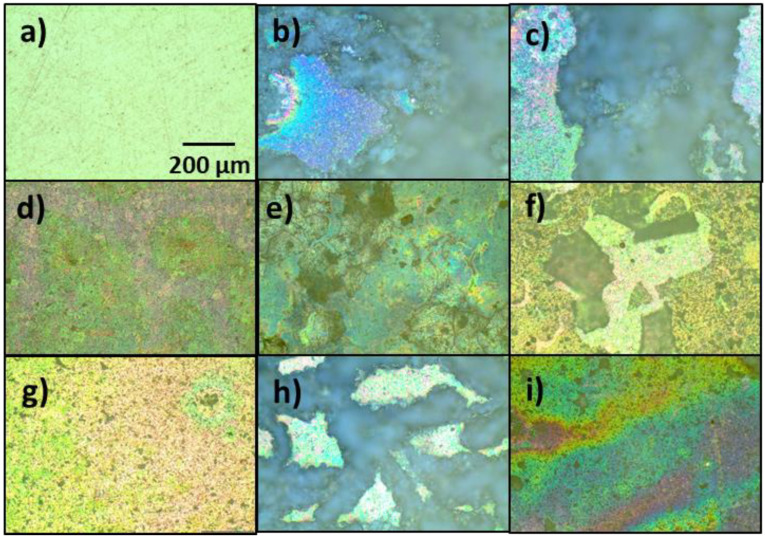
Optical microscopy images of the surfaces of the samples: (**a**) sample 0, uncorroded AZ61 alloy as received; (**b**) sample A, corroded AZ61 alloy after the Hank’s solution corrosion test; (**c**) corroded sample A-SG; (**d**) corroded sample A-SG-CIS; (**e**) corroded sample A-SG-DMG; (**f**) corroded sample A-SG-QUI; (**g**) corroded sample A-SG-AMIN; (**h**) corroded sample A-SG-GRA; and (**i**) corroded sample A-SG-TI.

Figure 4 shows the optical microscopy (OM) images of the surfaces of the samples. The AZ61 alloy used as a reference (as received) and not submitted to the HBSS corrosion test (sample 0, Figure 4a), showed a smooth polished surface with some streaks in random directions due to the polished effect.

Figure 4b–i shows the optical microscopy images of the samples tested in the HBSS. In general, all of them suffered some kind of deterioration after the test as identified by pH measurements. With the exception of sample A (Figure 4b), which was not coated, the rest maintained the coating with more or less cracks. Sample A lost its original luster and showed an uneven surface, chemically attacked with the presence of deposits. In turn, the sol-gel-coated sample without inhibitors (sample A-SG, Figure 4c) showed a moderate corrosion state with several cracks and pits. 

In the same line, the sol-gel coated samples doped with corrosion inhibitors can be divided into two groups: (i) the first group of samples corresponding to samples A-SG-CIS, A-SG-DMG, and A-SG-TI with most of the surface in good conditions, except for the edges of the alloy that were more corroded and cracked (Figure 4d,e,i); and (ii) the second group of samples with a generalized corrosion on the whole surface and cracks of different sizes. Big cracks partially detached from the alloy appeared on samples A-SG-QUI and A-SG-GRA (Figure 4f,h), and very thin small cracks adhered to the surface for the A-SG-AMIN coating were noted (Figure 4g). 

### 3.4. SEM Characterization

**Figure 5:**
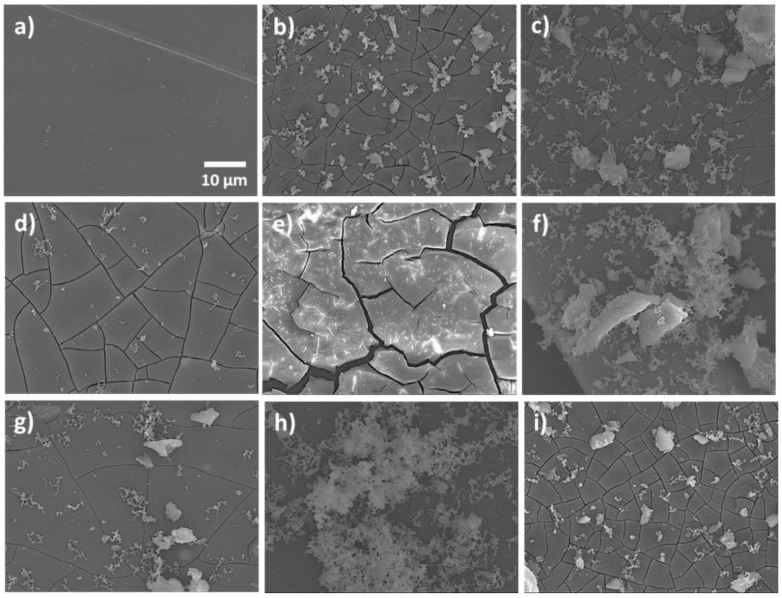
SEM images of the surfaces of the samples: (**a**) sample 0, uncorroded AZ61 alloy as received; (**b**) sample A, corroded AZ61 alloy after the HBSS corrosion test; (**c**) corroded sample A-SG; (**d**) corroded sample A-SG-CIS; (**e**) corroded sample A-SG-DMG; (**f**) corroded sample A-SG-QUI; (**g**) corroded sample A-SG-AMIN; (**h**) corroded sample A-SG-GRA; and (**i**) corroded sample A-SG-TI.

The surface morphologies of the samples were observed in detail by SEM in Figure 5, and EDX analyses were performed to verify the compositions of the compounds found, shown in Table 2. 

Sample 0, corresponding to the AZ61 alloy as received, presented a smooth surface with some scratches, most likely due to surface polishing as observed by optical microscopy (Figure 5a). Small white granular deposits were also observed on the surface of the sample. The EDX analysis of the surface indicated a composition similar to the nominal composition of the alloy [35]. In our case, Mg had 91 wt %, and Al had 5 wt %. There was also a residual 3% of the weight, which may be due to the oxidation of the alloy surface (Table 2). The local EDX analysis carried out on one of the white deposits on the surface showed that they were less soluble phosphorus salts and hydrated carbonates, most certainly coming from the HBSS. 

The surface of sample A (Figure 5b) had a deteriorated appearance with white deposits and cracks over its entire surface. The analysis of its surface showed a lower Mg content (61.8 wt %) compared to the unattacked alloy (91.6 wt %). The O content increased from 3.4 to 26.4 wt %, indicating a strong oxidation of the Mg in the alloy. The Al content remained more or less stable at around 5 wt % in both samples. This indicated that what reacted in the alloy was the Mg. The presence of Ca and P in this sample is due to poorly soluble salts from the HBSS that were not removed after the cleaning process with distilled water and remained adhered to the sample surface (Table 2).

The SEM images of sample A-SG showed a cracked surface with numerous deposits on its surface (Figure 5c). The crack spacing appeared to be narrower in this sample than in the uncoated sample. The EDX analyses showed the presence of a small amount of Si (0.5 wt %) and C (7.6 wt %), most likely due to the thin organic–inorganic hybrid coating layer that remained adhered to the surface of the sample (Table 2).

Sample A-SG-CIS showed a homogeneously cracked surface, with no detachment from the substrate alloy and some whitish deposits (Figure 5d). The EDX analyses indicated the presence of the sol-gel coating (2.5 wt % Si, 5.1 wt % C, and 29.8 wt % O). The high amounts of oxygen and phosphorus indicated the formation of phosphates, oxides, and hydroxides of magnesium or calcium due to the corrosion process (1.7 wt % Ca and 2.9 wt % P) (Table 2).

The surface of sample A-SG-DMG appeared cracked in SEM images, but the coating continued to be adhered to the surface. The area between the cracks was larger, and the cracks seemed to be deeper than in the case of the previous sample (Figure 5e). The EDX analysis of the surface showed higher amounts of Si (22.1 wt %) and C (32.3 wt %). The relative amounts of the alloy (the content of Mg: 3.8 wt %; the content of Al: 0.3 wt %) seemed to indicate that the coating was thicker than previous coated samples (Table 2).

Sample A-SG-QUI showed whitish deposits of insoluble salts of Hanks’ solution and areas of the partially detached coating on its surface (Figure 5f and Table 2). From the EDX analysis, the presence of calcium phosphates was deduced (5.3 wt % Ca and 3.4 wt % P), together with the sol-gel coating (4.0 wt % Si, 19.8 wt % C, and 35.4 wt % O) (Table 2).

Sample A-SG-AMIN (Figure 5g) showed a homogeneous surface with small cracks, less abundant than sample A. Some white deposits can also be appreciated (Figure 5g and Table 2). The EDX analysis indicated a high proportion of the alloy (62.9 wt % Mg and 4.8 wt % Al), so the surface would be less corroded but very oxidized (21.3 wt % O). Small amounts of Si and C from the sol-gel coating were also visible, as well as deposits of calcium phosphate salts from the Hanks’ solution.

The SEM images of sample A-SG-GRA showed uniform corrosion on the surface, with few cracks and abundant deposits of white salts (Figure 5h). The EDX analysis indicated the presence of Si, C, and O coming from the coating, traces of insoluble salts from the Hanks’ solution (Ca and P), and Mg and Al together with small amounts of Nb (2.8 wt %) and Zn (0.6 wt %) that came from the alloy composition (Table 2). These results are consistent with results from Gong et al. [36].

Sample A-SG-TI, including TiO_2_ NPs in the overlying sol-gel coating as a corrosion inhibitor, showed a cracked surface with white deposits. The cracks spacing of this sample seemed to be like in the uncoated corroded alloy (Figure 5i). No Si or C was observed in the EDX analysis, so the coating may have flaked off. The high amount of O (29.9 wt %) may indicate a high oxidation of the Mg–Al alloy, and Ca and P were also visible, indicating the presence of salt residues from the Hanks’ solution. Ti was also not observed in the analysis, because the amount added in the coating was 0.02% weight/volume and the thickness of the coating was around 100 nm, so the Ti content may be below the detection limit of the technique (Table 2).

From the results obtained here, in addition to the degradation of the AZ61 alloy during the Hanks’ solution corrosion test, we confirmed that all coatings degraded to a greater or lesser extent. However, appreciable differences were found in the different coatings prepared.

The uncoated alloy immersed in the Hanks’ solution for 14 days for the test suffered strong surface oxidation, suggested by the pH measurements. Fine cracks appeared, noticeable by both optical and scanning electron microscopies. 

The use of coatings with corrosion inhibitors improved the anticorrosion properties, with properties depending on the corrosion inhibitors incorporated in the coating. The corrosion inhibitor that seemed to work best was L-cysteine (Figure 2). The pH of the Hanks’ solution with the coated sample A-SG-CYS increased for the first two days, and then it maintained stable during the rest of 12 days. The total pH increased by only 0.75. In fact, the optical microscopy and SEM images showed similar behaviors to those of the uncoated sample with less cracks and crystalline deposits on its surface. 

Samples A-SG-DMG, A-SG-QUI, A-SG-AMIN, and A-SG-TI presented an increase of the pH during the Hanks’ solution corrosion test (Figure 2), cracks, and white deposits on their surfaces (Figure 4 and Figure 5). 

Sample A-SG-GRA showed an increase of the pH of the Hanks’ solution closer to the A-SG blank sample without corrosion inhibitors (Figure 2). Macroscopically, it showed partially detached cracks (Figure 4) and abundant white deposits on its surface (Figure 5). This behavior may be due to an inadequate proportion of the graphene nanosheets within the sol-gel coating. The EDX analysis also showed the presence of Nb and Zn ions, which were most likely the result of alloy degradation (Table 2). These minority components, due to their low solubility, appeared concentrated and deposited on the surface of Mg alloys [36].

The presence of the elements P and Ca in the EDX chemical analyses indicated the formation of insoluble calcium phosphate salts when the alloy was put in contact with Hanks’ solution simulating the biological fluid (Table 2). Their presence was related to the formation of hydroxyapatite (bone component) [37]. Therefore, an Mg–Al alloy with a coating that favored its growth formed more stable bonds and had better biocompatibility. The chemical and structural similarity of the alloy to be used as a biomaterial was greater in this case, creating optimal physicochemical conditions for the proliferation of bone cells, improving the physical, chemical, and biological activities of the implant. In our particular case, samples A-SG-QUI, A-SG-GRA, and A-SG-TI can have this characteristic, as they had high weights % of P and Ca on their surfaces (Table 2).

NPs added to the sol-gel hybrid coating did not perform a better corrosion effect than the ones containing molecules. OM(Optical microscopy) and SEM images appear more deteriorated than those samples that include molecules as corrosion inhibitors. For this reason, for corrosion protection in biological fluids we would say that molecules work better as protection. Among the molecules tested, sample A-SG-CIS shows the protection desirable for the AZ61 alloy in implant applications.

## 4. Conclusions

Mg alloys are good options to be used as biocompatible prostheses for humans, when these alloys are surrounded by biological fluid undergoing a certain degree of corrosion over time. In this work, we have prepared several hybrid sol-gel coatings doped with potential corrosion inhibitors as alternatives to improve the corrosion resistance of the AZ61 magnesium alloy.

The effectiveness of the hybrid sol-gel coatings with and without corrosion inhibitors has been evaluated by subjecting coated AZ61 magnesium alloy samples to a Hanks’ solution corrosion test by simulating the biological fluid during 14 days.

The results of this research showed that these sol-gel coatings were suitable to improve long-term performance of the AZ61 magnesium alloy. Coatings adhesion was good even without any kind of pre-treatment. After the Hanks’ solution corrosion test, sol-gel coatings were still adhered to the alloy surface. The pH of the solution of the non-coated alloy increased during the 14 days of the corrosion test, and a decrease of the pH was noticed for the coated sample.

After the Hanks solution corrosion test, the hybrid sol-gel coating doped with L-cysteine stood out from the others, keeping the pH of the solution constant after two days and thus indicating that a stabilization of the corrosion rate might take place. Other molecules such as dimethyl glyoxime, quinine, 2-Aminopyridine, or TiO_2_ NPs diminished the corrosion rate of the sol-gel coating, when they were introduced as corrosion inhibitors but underwent a certain degree of degradation over time.

Sol-gel coatings doped with NPs such as graphene sheets would not act well enough as corrosion inhibitors in the chosen synthesis conditions, but they seemed to promote the formation of calcium phosphates (maybe hydroxyapatite), thus improving the alloy biocompatibility. 

Although more extensive research is still needed, these results are promising for the use of Mg–Al alloys for its applications in the biomedical field.

## Figures and Tables

**Table 1:** Descriptions of the prepared samples.

Sample Name	Sol-Gel Coating at a TEOS:MEMO Ratio of 1:1 (*v*/*v*)	Corrosion Inhibitor Added	Corrosion Test Performed
0	No	No	No
A	No	No	Yes
A-SG	Yes	No	Yes
A-SG-CIS	Yes	L-cysteine	Yes
A-SG-DMG	Yes	Dimethyl glyoxime	Yes
A-SG-QUI	Yes	Quinine	Yes
A-SG-AMIN	Yes	2-Aminopyridine	Yes
A-SG-GRA	Yes	Graphene oxide	Yes
A-SG-TI	Yes	TiO_2_ nanoparticles	Yes

**Table 2:** Chemical compositions of the surfaces of the prepared samples performed by EDX.

Chemical Composition (wt %)	Samples								
	0	A	A-SG	A-SG-CIS	A-SG-DMG	A-SG-QUI	A-SG-AMIN	A-SG-GRA	A-SG-TI
Mg	91.6 ± 0.7	61.8 ± 1.0	51.6 ± 1.4	51.6 ± 0.4	3.8 ± 0.1	27.5 ± 0.4	62.9 ± 0.8	53.1 ± 0.7	50.6 ± 1.0
Al	5.0 ± 0.4	4.7 ± 0.4	3.9 ± 0.3	5.8 ± 0.1	0.3 ± 0.1	2.3 ± 0.1	4.8 ± 0.2	3.5 ± 0.1	4.1 ± 0.4
Si	-	-	0.5 ± 0.2	2.5 ± 0.1	22.1 ± 0.4	4.0 ± 0.1	0.2 ± 0.1	0.5 ± 0.1	-
C	-	-	7.6 ± 2.0	5.1 ± 0.6	32.3 ± 1.0	19.8 ± 1.0	5.8 ± 1.0	4.7 ± 0.9	-
O	3.4 ± 0.6	26.4 ± 1.0	28.7 ± 1.2	29.8 ± 0.3	40.7 ± 0.8	35.4 ± 0.7	21.3 ± 0.5	26.0 ± 0.5	29.9 ± 1.2
Ca	-	3.2 ± 0.2	4.0 ± 0.3	1.7 ± 0.1	0.8 ± 0.1	5.3 ± 0.1	1.9 ± 0.1	4.4 ± 0.1	7.5 ± 0.4
Cl	-	-	-	-	-	-	0.2 ± 0.1	-	-
P	-	3.9 ± 0.4	3.7 ± 0.4	2.9 ± 0.1	-	3.4 ± 0.1	2.2 ± 0.1	4.4 ± 0.1	7.9 ± 0.5
Nb	-	-	-	-	-	2.3 ± 0.4	-	2.8 ± 0.4	-
Zn	-	-	-	0.6 ± 0.1	-	-	0.7 ± 0.2	0.6 ± 0.2	-
